# Challenges and Opportunities in Implementing Digital Technology in Dental Curriculum: A Review and Perspective

**DOI:** 10.7759/cureus.83272

**Published:** 2025-04-30

**Authors:** Hiroe Ohyama, Mai-Ly Duong, Aaron E Yancoskie, Alayna B Smiley, Ali Z Syed, Michael S Reddy, Sompop Bencharit

**Affiliations:** 1 Restorative Dentistry and Biomaterials Sciences, Harvard School of Dental Medicine, Boston, USA; 2 General Dentistry, Arizona School of Dentistry and Oral Health, A.T. Still University, Mesa, USA; 3 Oral and Maxillofacial Pathology, Touro College of Dental Medicine, New York Medical College, New York City, USA; 4 Oral and Maxillofacial Surgery, Greater Washington Oral and Maxillofacial Group, International Implant Institute, Fairfax, USA; 5 Oral Medicine and Diagnostic Sciences, Case Western Reserve University School of Dental Medicine, Cleveland, USA; 6 Periodontology, University of California San Francisco, San Francisco, USA; 7 Prosthodontics, Workman School of Dental Medicine, High Point University, High Point, USA

**Keywords:** dental education, dental innovation, dental technology, digital dentistry, educational research

## Abstract

Digital dentistry technology has ushered in a transformative era in the field of dentistry, offering a myriad of compelling benefits that encompass enhanced patient care, elevated diagnostic capabilities, and vastly more efficient workflows. Dental schools, as the vanguards of dental education, find themselves on the front lines of adopting and integrating these advanced technologies into their pedagogical frameworks and clinical practices. This literature review comprehensively explores the myriad of challenges and issues encountered by dental schools as they embark on the implementation journey of digital dentistry technology, shedding light on the implications for institutions, educators, students, and patients. Digital dentistry encompasses a wide array of technological innovations, such as intraoral scanners, digital radiography, 3D imaging, CAD/CAM systems, and electronic health records (EHRs). These innovations have redefined the way dentists diagnose, plan treatment, and provide care to their patients. Dental schools are embracing these technologies to remain relevant and effective in educating the next generation of dentists. This review delves into the multifaceted challenges they confront, spanning educational, financial, technological, psychological, ethical, and regulatory dimensions, through research and review articles, textbooks, and case studies. The integration of digital dentistry into curricula demands a reevaluation of content, pedagogy, faculty development, and infrastructure. The financial burden associated with acquiring and maintaining digital equipment poses significant challenges, requiring meticulous financial planning. Resistance to change from faculty, staff, and students necessitates effective change management and communication strategies. Standardization, calibration, patient acceptance, and privacy concerns, along with regulatory and accreditation compliance, require rigorous attention. These challenges hold far-reaching implications for the dental profession, affecting education, patient care, and the adaptability of the dental workforce in a technology-driven era. This review examines each challenge in depth, drawing from empirical evidence and worldwide dental school experiences. It provides valuable insights for dental educators, administrators, students, and stakeholders to navigate these complexities effectively, ensuring the successful integration of digital dentistry technology into dental education and practice. In conclusion, while digital dentistry technology promises substantial benefits, addressing the associated challenges is paramount. Dental schools must strategically allocate resources, invest in faculty and student training, foster a culture of adaptability, and adhere to regulatory and accreditation standards. Successfully navigating these challenges will empower dental schools to prepare graduates who can provide high-quality, technology-driven patient care, ultimately advancing the field of dentistry in the digital age.

## Introduction and background

Digital dentistry technology has transformed dentistry by offering promising benefits that encompass enhanced patient care, elevated diagnostic capabilities, and vastly more efficient workflows [1-4]. As the vanguards of dental education and the nurturing grounds for the dental professionals of tomorrow, dental schools find themselves on the front lines of adopting and integrating these advanced technologies into their pedagogical frameworks and clinical practices [5-8]. This literature review seeks to undertake a comprehensive exploration of the challenges encountered by dental schools as they embark on the implementation journey of digital dentistry technology. In doing so, it aims to shed light on the far-reaching implications of these challenges, not only for the institutions themselves but also for the educators, students, and patients directly affected by this paradigm shift in dental education and practice.

Digital dentistry, as a collective term, encompasses a wide array of technological innovations and advancements that have significantly altered the landscape of dental care and education in recent years. These innovations include, but are not limited to, intraoral scanners, digital radiography, 3D imaging, computer-aided design and computer-aided manufacturing (CAD/CAM) systems, and electronic health records (EHRs) [9]. Collectively, they have redefined the way dentists diagnose, plan treatment, and provide care to their patients [10]. The benefits of these technologies are well-documented in the literature, where studies and reviews have consistently demonstrated their capacity to improve diagnostic accuracy, enhance treatment outcomes, reduce treatment times, and ultimately elevate the quality of care provided as well as forensic analysis [1-5,11]. Additionally, the use of digital technologies has shown great promise in aiding faculty and students in the assessment of preclinical projects in undergraduate dental education [12-14]. The emergence of VR-Haptics technology as a tool to simulate clinical situations has been expanding globally [15,16].

Dental schools in the United States have increasingly embraced CAD/CAM technology, especially in fixed prosthodontics, yet the adoption remains uneven and continues to face notable challenges. Most predoctoral programs introduce CAD/CAM concepts through lectures (87.2%) and simulated exercises (86.9%), with instruction predominantly provided by faculty (87.9%). Hands-on learning is supported through video demonstrations (81.8%), small-group demos (69.2%), and workshops (65.6%), yet less than half of the students reported using intraoral scanners in clinical settings (45.2%). While students’ knowledge and attitudes toward CAD/CAM improve as they advance in their programs, their satisfaction with CAD/CAM education remains neutral, signaling a need for curricular reform [17]. Digital removable prosthodontics, particularly CAD/CAM complete and partial dentures, lags further behind. Only 54.2% of predoctoral and 65.2% of advanced graduate programs include CAD/CAM complete dentures in didactic courses, and CAD/CAM removable partial dentures are taught in just 37.5% of predoctoral and 47.8% of graduate programs. Financial constraints, limited faculty expertise, and time within the curriculum are persistent barriers to broader integration. Despite growing enthusiasm among students and faculty alike, these findings underscore the urgent need for targeted investments, faculty training, and research to fully realize the potential of digital technologies across prosthodontic education [18].

Although the advantages of digital dentistry are clear, the literature offers limited insight into how these technologies are effectively integrated into dental school curricula [1-8]. There is a noticeable lack of prospective studies, unified frameworks, and comparative evaluations of implementation models across institutions. Existing reports often focus on individual technologies or case examples without addressing broader systemic barriers, such as institutional readiness, faculty adoption, and student preparedness. Moreover, few studies explore the long-term educational and clinical outcomes of digital integration [11-14].

Historically, digital dentistry traced back to 1976 when Francois Dure presented the idea of CAD/CAM dentistry; however, it began to gain traction in clinical practice during the early 2000s with the wider availability of CAD/CAM systems and digital radiography [1-8]. However, its educational integration has been slower and more fragmented. As technological innovation continues to accelerate, particularly with the emergence of artificial intelligence (AI), the pressure on academic institutions to adapt has intensified [19]. Fast-emerging implementations of diagnostic tools such as intraoral scanning in caries diagnosis [20,21] and radiographic periodontal disease monitoring are now integrated with AI [22-24]. Yet, there remains a gap in understanding how these changes are being managed in different educational environments.

Dental schools, as pivotal institutions in shaping the future of dentistry, have a dual responsibility: to equip their students with the knowledge and skills required to excel in a technology-driven profession and to serve as incubators for research and innovation in dental care. Consequently, these institutions have embarked on a journey to embrace digital dentistry technologies in order to remain relevant and effective in educating the next generation of dentists [5-8]. In this context, this review endeavors to illuminate the multifaceted challenges and issues that dental schools inevitably confront during their endeavors to implement digital dentistry technologies. These challenges traverse various dimensions, including but not limited to educational, financial, technological, psychological, ethical, and regulatory aspects. Each of these dimensions poses a unique set of hurdles and requires careful navigation. This review will dissect these challenges in-depth, drawing on both empirical evidence and anecdotal experiences from dental schools worldwide.

The success or failure of digital dentistry technology implementation in dental schools reverberates across the entire dental profession [5-8]. As we delve into the core of this literature review, we will navigate through the intricate web of challenges and issues, each deserving meticulous examination and analysis. By the end of this comprehensive exploration, we hope to provide dental educators, administrators, students, and policymakers with valuable insights into the complexities of implementing digital dentistry technology in dental schools. These insights, we anticipate, will serve as a roadmap to facilitate more effective navigation of the challenges and, ultimately, ensure the successful integration of digital dentistry technology into dental education and practice.

## Review

Literature search

A narrative review was conducted to examine the implementation and integration of digital dentistry in dental schools worldwide. A literature search was performed in PubMed, Scopus, and Web of Science for articles published between January 2014 and April 22, 2025. Search terms included “digital dentistry,” “dental education,” “CAD/CAM,” “intraoral scanners,” “3D printing,” and “curriculum integration.”

Articles were included if they focused on the adoption of digital dentistry technologies in predoctoral or postgraduate dental programs. Studies unrelated to educational settings or focused solely on technical development were excluded. Relevant literature was selected through title and abstract screening, followed by full-text review.

Curricular integration of technology

An essential yet often overlooked factor in the successful implementation of digital dentistry in dental schools is institutional leadership. Buy-in must begin at the top, specifically with the Dean and senior administration, who ultimately shape the strategic vision and allocate resources for curriculum development and technological integration. The adoption of digital systems requires substantial investment, not only in equipment but also in faculty development, infrastructure, and workflow redesign. Without strong leadership to champion the initiative, even the most advanced tools may remain underutilized. Moreover, the ability to mobilize faculty engagement, secure funding, and align institutional priorities with evolving educational standards depends heavily on this top-down commitment. Therefore, leadership plays a pivotal role in determining whether digital transformation efforts are fragmented and temporary or strategic and sustainable [15-17].

The integration of digital dentistry technology into existing curricula poses a fundamental challenge for dental schools, necessitating a comprehensive overhaul of educational approaches and pedagogical methods [5,13,15-22]. This transformation is driven by the incorporation of advanced digital tools, such as CAD/CAM systems and intraoral scanners, which have the potential to revolutionize dental education and practice [5,13,24-35].

The adaptation of curricula to accommodate digital dentistry technology requires a multifaceted approach. Some of the key dimensions of this challenge are mentioned in Table 1.

**Table 1:** Key dimensions in technology integration challenges Case report/technical report [26]; narrative review/perspective paper [7,20,24,29]; scoping review/systematic review/meta-analysis [16,19,23]; and research article (for educational methodology/survey) [30].

Key area	Description
Content reevaluation	Traditional curricula emphasize manual techniques; digital tools require curriculum updates to include digital skills in diagnosis, treatment planning, restorative dentistry, and patient management [26,29]
Pedagogical transformation	Teaching strategies must evolve to include digital learning resources, virtual patient cases, and simulation exercises for hands-on experience with digital tools [29]
Faculty Development	Faculty must gain proficiency in digital technologies, requiring investment in training programs for CAD/CAM, intraoral scanners, digital radiography, and treatment planning software [20,29]
Infrastructure and resources	Schools need to invest in digital equipment, software, and dedicated digital dental labs, ensuring financial planning for sustainability [30]
Curricular horizontal and vertical integration	Digital dentistry should be incorporated across preclinical, laboratory, and clinical courses, requiring interdepartmental coordination. [23,24,26]
Assessment and competencies	Schools must develop assessment methods aligned with digital dentistry competencies to ensure students' proficiency in clinical application [7,16,19]
Continuous adaptation	Digital dentistry evolves rapidly; curricula must be regularly updated, requiring ongoing faculty training and an innovation-driven culture [26]

The successful integration of digital dentistry technology into dental school curricula requires a strategic and multidimensional approach that demands significant investments in faculty development, infrastructure, content reevaluation, and pedagogical transformation. Dental educators and administrators must collaborate and commit to a dynamic and forward-thinking approach to ensure that students graduate with the digital competencies necessary for modern dental practice. Ultimately, this transformative journey holds the promise of preparing dental professionals who are well-equipped to provide high-quality, technology-driven patient care.

Cost and infrastructure

The financial aspect of implementing digital dentistry technology in dental schools is a crucial consideration that warrants a detailed examination. The adoption of state-of-the-art digital dental equipment necessitates significant financial investments, constituting a substantial burden for dental schools [9,10,31]. 

At the outset, the high initial costs associated with procuring cutting-edge digital dental equipment can be staggering. These costs encompass the purchase of intraoral scanners, 3D imaging systems, CAD/CAM machines, digital radiography units, and EHR systems, among others. While these technologies offer long-term benefits, the initial capital outlay can strain the financial resources of dental schools, particularly those with limited budgets. Allocating funds for the purchase of digital equipment often necessitates careful financial planning and may require schools to reallocate resources from other areas.

Dental schools face substantial financial burdens when integrating digital dentistry technologies, due to both high upfront costs and ongoing maintenance expenses. For example, intraoral scanners can range from $15,000 to $40,000 each, depending on the brand and included software. CAD/CAM systems, which include design software and milling units, can cost between $80,000 and $150,000 per unit. Cone-beam computed tomography (CBCT) machines used for 3D imaging may range from $90,000 to over $250,000. Digital radiography units can add another $10,000-$30,000 per operatory. High-resolution dental 3D printers used for printing models, surgical guides, dentures, and more can cost between $5,000 and $25,000, with some high-end or production-grade units exceeding $50,000. Beyond equipment acquisition, schools must budget for recurring costs such as software licenses, calibration services, resins and printing materials, technical support, and hardware upgrades. To manage these expenses, dental schools can create sustainable financial models by phasing equipment purchases over several budget cycles, securing vendor partnerships with educational discounts, and applying for grants focused on digital innovation and healthcare technology. Additionally, schools can offset some of the cost by reducing reliance on traditional lab services, streamlining workflow efficiencies, or expanding digital-based clinical services, which may enhance productivity and potentially increase revenue. Including long-term replacement and upgrade plans in institutional budgeting ensures that technology remains current while avoiding the financial shock of sudden obsolescence. Strategic return-on-investment (ROI) planning can further demonstrate the value of digital integration by linking it to improved educational outcomes and patient care quality, thus reinforcing administrative and stakeholder support.

Beyond the initial investment, dental schools must contend with ongoing maintenance expenses. Digital dental equipment, like any technology, requires regular maintenance and calibration to ensure accuracy and reliability. Maintenance costs encompass servicing, software updates, and the replacement of components that wear out over time. Ignoring or neglecting maintenance can result in equipment downtime, compromised patient care, and increased long-term costs. Consequently, dental schools must allocate budgetary resources and establish sustainable financial models to cover these ongoing maintenance expenses.

Furthermore, the fast-paced evolution of digital dentistry technology means that periodic updates and upgrades are inevitable. Dental schools must anticipate and budget for these updates to remain current and competitive. Falling behind in technology can hinder the quality of education and patient care provided, potentially affecting the reputation of the institution.

In addition to equipment costs, dental schools must address infrastructure challenges. The seamless operation of digital dental equipment relies heavily on a robust IT network and appropriate facilities [1,4,32]. Dental schools must invest in the necessary infrastructure to support these technologies effectively. This entails ensuring high-speed and reliable network connectivity, implementing data storage solutions, and creating dedicated spaces for digital workflows.

Infrastructure investments extend beyond IT considerations to encompass facility modifications. Specialized operatories and laboratory spaces may be required to accommodate digital equipment and workflows. These spaces must be designed with ergonomics, infection control, and workflow efficiency in mind.

Moreover, the integration of digital dental technology into the curriculum necessitates changes in teaching spaces and methodologies. For example, a modernized fixed prosthodontics course would necessitate the integration of intraoral scanners, prosthetic design software, CAD workstations, milling units, 3D printers, ceramic sintering, and characterization furnaces, along with post-processing systems to support the complete digital workflow [16,17,25,26]. Similarly, a contemporary removable prosthodontics course would require digital impression systems, software for designing complete and partial dentures, 3D printers capable of printing denture bases and teeth, as well as equipment for post-processing and polymerization. In implantology, the curriculum would need to include cone-beam computed tomography (CBCT) units, digital planning software for implant positioning, guided surgery tools, CAD/CAM systems for custom abutments and prostheses, and simulation technologies for surgical training [16,19,25,26,29]. Dental schools may need to invest in digital learning resources, such as virtual reality simulation tools and digital textbooks, to enhance the educational experience for students.

The financial challenges associated with adopting and maintaining digital dentistry technology in dental schools are multifaceted and significant [26]. Addressing these challenges requires meticulous financial planning, sustainable budgeting models, and the ability to adapt to the evolving nature of technology. While the initial costs and ongoing expenses can be substantial, the potential benefits, including improved education and patient care, make these investments imperative for dental schools committed to staying at the forefront of dental practice and education [12,13]. The dental school administration may be mindful in developing a sustainable plan by leveraging the reduction of lab costs to help offset the initial investment and to keep maintaining the equipment. Moreover, a long-term replacement and upgrading plan for any hardware and software should also be considered. When the ROI is well-planned, there is a potential to expand clinical operations and increase productivity as well as pass along fee reduction to patients [21]. Successful financial management in the context of digital dentistry implementation is vital for ensuring that dental schools can harness the full potential of these technologies while maintaining their financial stability and long-term viability (Table 2).

**Table 2:** Key financial considerations Case report/technical report [26,34]; narrative review/perspective paper [29,35,36]; and research article (for educational methodology/survey) [27,33]. ROI: return-on-investment

Key financial factor	Description
Initial investment	High upfront costs for purchasing intraoral scanners, CAD/CAM machines, 3D imaging systems, and digital radiography units require strategic financial planning [27,33,35]
Ongoing maintenance	Regular servicing, software updates, and calibration are necessary to ensure equipment reliability and accuracy, requiring sustained budget allocation [29]
Technology upgrades	Continuous advancements in digital dentistry necessitate periodic updates and upgrades to remain competitive and maintain high-quality education and patient care [26]
IT infrastructure	Investment in high-speed networks, data storage, and secure systems is essential for seamless digital workflow and operational efficiency [26,29]
Facility modifications	Specialized operatories and labs must be designed to accommodate digital workflows, considering ergonomics, infection control, and teaching methodologies [34]
Sustainable budgeting	Schools must develop long-term financial plans, potentially offsetting costs by reducing lab expenses and leveraging ROI from enhanced clinical operations [29,36]
Financial planning for ROI	A well-structured investment plan can expand clinical services, increase efficiency, and pass cost savings to patients while maintaining financial stability [29,36]

Training and skill development

Specialized training for dental students and faculty members is a pivotal component of the successful integration of digital dentistry tools into dental education and practice [16]. These digital tools encompass a wide range of technologies, thus proficiency in using these tools is essential for providing high-quality patient care in the modern dental landscape (Table 3). Developing the necessary skills to operate and interpret digital images, design and fabricate restorations digitally, and manage digital patient records can indeed be challenging and time-consuming [31]. The complexity of these tools, combined with the need for precision and attention to detail, necessitates structured training programs that can bridge the gap between traditional dental education and the demands of digital dentistry. To address these challenges and ensure that students and faculty are adequately prepared to leverage digital technology, dental schools must make strategic investments in training and skill development [7,16,32-38]. 

**Table 3:** Considerations for developing technology skills and expertise Case report/technical report [26,31]; narrative review/perspective paper [29,35]; scoping review/systematic review/meta-analysis [38]; and research article (for educational methodology/survey) [25,27,30].

Key area	Description
Comprehensive training programs	Dental schools should integrate theoretical and practical digital dentistry training into the curriculum, tailored to different educational levels [29]
Hands-on workshops	Workshops and simulation exercises provide students and faculty with practical experience in intraoral scanning, digital impressions, CAD/CAM restoration design, and 3D printing [27,29]
Faculty development	Faculty training programs are essential to ensure educators are proficient in digital dentistry technologies and can effectively teach their application [25,26,27,29,30,31]
Access to resources	Schools must provide access to digital equipment, software, and educational materials, potentially through dedicated digital dental labs or clinics [29]
Continuous learning	Given rapid technological advancements, ongoing training, workshops, and updates on emerging digital dentistry technologies should be encouraged for students and faculty [35]
Assessment of competencies	Schools should implement competency assessments using multiple evaluation methods to ensure students achieve proficiency in digital tools [35]
Interdisciplinary collaboration	Collaboration among dental disciplines (e.g., prosthodontics, orthodontics, and oral surgery) enriches digital dentistry training by simulating real-world patient care scenarios [35,38]

Digital designing enhances dental education by adding powerful visualization tools and opportunities for real-life clinical simulation. Through CAD software, students can visualize tooth anatomy, occlusion, and spatial relationships in ways that are not easily achievable with traditional methods. This digital approach allows for precise planning and real-time modifications, making it an effective tool for teaching clinical decision-making and restorative principles. In contrast, traditional techniques such as wax-ups and tooth setups remain valuable for developing hand skills and tactile understanding of form and function. Together, these approaches create a balanced educational experience: digital tools provide efficient, repeatable design experiences and clinical relevance, while traditional methods foster manual dexterity and a deep appreciation of dental morphology. Integrating both methods allows dental students to build a strong foundation in both the artistic and technological aspects of modern dentistry [18,33-38]. 

 Dental schools must commit resources, time, and expertise to ensure that both students and faculty members are proficient in using digital tools effectively. By doing so, dental schools can empower their graduates to provide state-of-the-art, technology-driven patient care and contribute to the advancement of the field.

Resistance to change

Resistance to change is a pervasive and formidable challenge that dental schools encounter when striving to implement digital dentistry technology [5,16]. This resistance can emanate from various stakeholders within the educational ecosystem, including faculty members, staff, and even students, and often originates from a complex interplay of factors (Table 4).

**Table 4:** Addressing challenges and resistance Narrative review/perspective paper [29,36] and research article (for educational methodology/survey) [35].

Key area	Description
Engage stakeholders	Involving faculty, staff, and students in decision-making fosters a sense of ownership and commitment to digital dentistry adoption [35]
Education and training	Comprehensive training programs help demystify digital technologies, providing resources and support to build competence [36]
Pilot programs	Small-scale pilot programs allow gradual acclimation to digital tools and provide opportunities for feedback and improvement [29]
Change champions	Identifying faculty or staff members enthusiastic about digital dentistry can inspire others and offer peer support [29]
Clear communication	Transparent communication about the rationale, benefits, and timeline of digital dentistry implementation helps mitigate uncertainty [36]
Resource allocation	Ensuring financial and human resources are available for a smooth transition, including technical support and troubleshooting, is crucial [36]
Evaluation and feedback	Continuous assessment and stakeholder feedback help refine and improve digital dentistry integration over time [36]

One of the primary drivers of resistance to change is the perceived learning curve associated with adopting new digital technologies [37]. Faculty members, many of whom have been accustomed to traditional teaching methods and tools, may express concerns about their ability to adapt to digital platforms effectively. Staff members responsible for managing and maintaining digital equipment may also encounter challenges in acquiring the necessary skills. Moreover, students, though often considered tech-savvy, may have varying levels of comfort and familiarity with digital dentistry tools, leading to apprehension about the transition.

Disruption of established routines represents another significant source of resistance [38]. Dental schools, like many institutions, tend to adhere to established practices and routines that have evolved over time. The introduction of digital dentistry can disrupt these routines, necessitating changes in teaching methodologies, clinical workflows, and administrative processes. Faculty members and staff may be concerned about potential disruptions to their daily tasks and responsibilities.

Faculty members in dental schools face increasing challenges in keeping pace with the rapid advancement of technologies such as intraoral scanners, CAD software, 3D printers, evolving digital materials, and most notably, artificial intelligence (AI). Daily responsibilities now often require proficiency in operating and troubleshooting complex digital systems, staying updated with frequent software updates and new workflows, and ensuring the compatibility and integration of various platforms across clinical and preclinical settings. Faculty must also critically evaluate AI-driven diagnostic tools and automated design features, which can raise questions about clinical accuracy, oversight, and ethical implications. Balancing these tasks with existing teaching, mentoring, and research obligations can be overwhelming, especially for those trained in traditional methods. Additionally, the expectation to continuously adapt and redesign course content to reflect technological changes places added pressure on educators to not only learn these tools but also to teach them effectively within already packed curricula [18,28,30,32-38].

Fear of technological obsolescence is a genuine concern among stakeholders [37]. In the rapidly evolving landscape of digital dentistry, the fear that newly acquired skills and knowledge may become outdated quickly can hinder enthusiasm for change. Faculty members and even students may worry that the technology they invest time and effort in learning may become obsolete in a short period.

To overcome resistance to change in the context of digital dentistry implementation, dental schools must employ effective change management strategies and prioritize clear and open communication [18].

Standardization and calibration

The maintenance of accuracy and consistency in digital systems, particularly digital radiography and 3D imaging, is paramount in the realm of digital dentistry [8,16,33,38]. These technologies are essential for precise diagnosis, treatment planning, and patient care. To ensure reliable clinical outcomes, dental schools must establish rigorous protocols for system calibration, quality assurance, and standardization of procedures (Table 5).

**Table 5:** Important components in standardization and calibration Narrative review/perspective paper [29,34,36].

Key area	Description
Calibration protocols	Well-defined calibration protocols should be established for all digital imaging and diagnostic equipment. Regular calibration ensures accuracy in radiographic devices, intraoral scanners, and CAD/CAM systems [29,34,36]
Quality assurance	A comprehensive quality assurance program should be implemented, including periodic equipment checks, validation of imaging accuracy, and performance benchmarks to maintain consistent digital system standards [29,34,36]
Standardized procedures	Standardized protocols for image acquisition, processing, and interpretation should be promoted to enhance diagnostic reliability. These procedures should be integrated into the curriculum to ensure students learn best practices [36]

Patient acceptance and privacy

Patient acceptance and privacy concerns are critical considerations when integrating digital dentistry technology into dental practices [4,5,16,33]. Patients may have apprehensions about data security, privacy breaches, and the use of digital records. Addressing these concerns is vital to build trust and foster acceptance of digital technologies. 

Delivery of dental care has and will continue to rapidly involve technology. While patients have historically been accepting of the transition from paper records to digital records, the utilization of intra-oral scanning, teledentistry, and 3D software may not be as easy to obtain acceptance and satisfaction. Dental schools should acknowledge the increased risk for patient concerns regarding information breaches as well as increased concern for the accuracy and quality of dentistry delivered [39-41]. 

Dental schools should implement robust data protection measures to safeguard patient information. This includes encryption, secure storage, access controls, and regular cybersecurity assessments. Ensuring compliance with data protection regulations, such as the Health Insurance Portability and Accountability Act (HIPAA) in the United States, is essential [42-45].

Obtaining informed consent from patients is a fundamental ethical practice. Dental schools should teach students the importance of obtaining explicit consent for the use of digital technologies in diagnosis and treatment. Educating patients about how their data will be used and protected can alleviate concerns [45-49]. Dental schools have a role in educating patients about the benefits of digital dentistry technology. Patients should be informed about how digital tools enhance diagnostic accuracy, reduce radiation exposure, and improve treatment outcomes (Table 6). Transparent communication can help allay fears [48,49]. 

**Table 6:** Patient's factors Narrative review/perspective paper [29,35,45].

Key area	Description
Patient acceptance	While patients have adapted to digital records, acceptance of intraoral scanning, teledentistry, and 3D software may be more challenging. Dental schools should address concerns about data security and treatment accuracy [45]
Data protection measures	Robust data protection measures, including encryption, secure storage, access controls, and cybersecurity assessments, should be implemented to safeguard patient information and comply with regulations like HIPAA [45]
Informed consent	Dental schools should emphasize the importance of obtaining explicit patient consent for digital technology use in diagnosis and treatment, ensuring transparency in data usage and protection [45]
Patient education	Educating patients on the benefits of digital dentistry, such as improved diagnostic accuracy, reduced radiation exposure, and better treatment outcomes, can help alleviate concerns and build trust [29,35,45]

Regulatory and accreditation compliance

Dental schools must navigate regulatory requirements and accreditation standards when implementing digital dentistry technology. Compliance with relevant guidelines, such as those set by the Commission on Dental Accreditation (CODA) in the United States, is essential to ensure that educational programs meet the necessary standards. 

In regard to CODA standards for predoctoral education, while there is no single standard specifically dedicated to digital dentistry, several elements within Standards 2-24 (student education), 3-2 (faculty), and 5-2 (patient care), as well as statements pertaining to the educational environment, emphasize the incorporation of current and emerging technologies. However, there is no specific guidance on how the integration of additional new and emerging technologies may influence or replace traditional techniques in predoctoral education [50]. In contrast, for graduate programs such as prosthodontics, CODA explicitly requires comprehensive instruction in digital dentistry technologies. This includes Standard 4-11, which mandates in-depth instruction on digital dentistry; Standard 4-26, which requires residents to apply digital technologies in diagnosis, treatment planning, and prosthetic care; and Standard 4-33, which calls for instruction in advanced digital technology for craniofacial and prosthetic rehabilitation [51]. It is highly likely that, in the future, CODA will expand regulations related to digital dentistry at the predoctoral level as well as across other specialty training programs due to the increasing use of digital technology [52,53].

Navigating regulatory requirements and accreditation standards is a non-negotiable aspect of implementing digital dentistry technology in dental schools [24,25,38]. Compliance with these guidelines, which may vary by region and institution, is crucial to ensure that educational programs meet the necessary standards and maintain their accreditation status.

Dental schools must stay informed about the regulatory landscape governing digital dentistry in their region. This includes understanding requirements related to equipment certification, data security, and patient consent. Dental schools seeking accreditation, such as from the CODA in the United States, must align their digital dentistry practices with the relevant accreditation standards. This may involve demonstrating compliance with specific educational and clinical requirements related to digital technologies. Ongoing monitoring and assessment of compliance with regulations and accreditation standards are essential. Dental schools should establish mechanisms for periodic reviews, updates to policies and procedures, and documentation of compliance efforts (Table 7).

**Table 7:** Regulatory considerations Narrative review/perspective paper [45,50,51].

Key area	Description
Awareness of regulations	Dental schools must stay updated on regulations governing digital dentistry, including equipment certification, data security, and patient consent requirements [45]
Accreditation standards	Schools seeking accreditation (e.g., CODA in the U.S.) must ensure their digital dentistry practices align with relevant educational and clinical accreditation standards [50,51]
Continuous monitoring	Establishing mechanisms for periodic reviews, policy updates, and documentation of compliance efforts ensures ongoing adherence to regulations and accreditation requirements [45]

Standardization and calibration, patient acceptance and privacy, and regulatory and accreditation compliance are crucial aspects of the successful implementation of digital dentistry technology in dental schools. By prioritizing these considerations, dental schools can ensure the integrity of their educational programs, protect patient interests, and meet the evolving standards of the dental profession in the digital age.

Literature review limitations

The literature on digital technology integration in dental schools remains limited, with little research and few comprehensive reports addressing its full impact. While shared curricula exist through organizations such as the American Dental Education Association (ADEA) and the American College of Prosthodontists (ACP), these primarily focus on curricular content rather than broader implementation factors such as infrastructure, faculty development, and financial sustainability. Additionally, there is a lack of clear information on patient acceptance of digital dentistry, leaving uncertainties about how patients perceive and adapt to these advancements. The available studies on faculty and staff engagement are limited and often speculative, lacking standardized measures to assess readiness, training needs, and long-term adoption. To address these gaps, more prospective studies are needed to provide empirical data on the effectiveness, challenges, and long-term outcomes of digital technology integration in dental education.

## Conclusions

The integration of digital dentistry technology into dental school curricula and clinical practice offers significant opportunities but also presents complex challenges. To achieve successful implementation, dental schools should adopt a strategic, multifaceted approach. This includes systematically embedding digital workflows into didactic, preclinical, and clinical education; prioritizing faculty development through targeted training initiatives; allocating funding for infrastructure and technology upgrades; and fostering a culture that embraces innovation and adaptability. Institutions must also establish clear protocols for standardization and regulatory compliance and actively engage students and patients to promote acceptance of digital technologies. By taking these actionable steps, dental schools can ensure that graduates are proficient in digital competencies and confident in delivering high-quality, technology-driven, patient-centered care. Furthermore, there is a critical need for more longitudinal studies, implementation case studies, and comparative analyses across institutions to better inform best practices and guide future curricular advancements.
